# Novel *PSEN1* G209A mutation in early-onset Alzheimer dementia supported by structural prediction

**DOI:** 10.1186/s12883-016-0591-6

**Published:** 2016-05-20

**Authors:** Seong Soo A. An, Eva Bagyinszky, Hye Ryoun Kim, Ju-Won Seok, Hae-Won Shin, SeunOh Bae, SangYun Kim, Young Chul Youn

**Affiliations:** College of Bionano Technology, Gachon Bionano Research Institute, Gachon University, Seongnam-si, South Korea; Department of Laboratory Medicine, Chung-Ang University College of Medicine, Seoul, South Korea; Department of Nuclear Medicine, Chung-Ang University College of Medicine, Seoul, South Korea; Department of Neurology, Chung-Ang University College of Medicine, Chung-Ang University Hospital, 224-1 Heukseok-dong, Dongjak-Gu, Seoul, 06973 South Korea; Department of Neuology, Seoul National University Bundang Hospital and Seoul National University College of Medicine, 300 Gumidong, Bundang-gu, Seongnam-si, , Gyeonggi-do 463-707 South Korea

**Keywords:** Alzheimer's disease, Presenilin 1 mutation, Presenilin 1 protein structure, Novel mutation, Structural prediction

## Abstract

**Background:**

Three main genes are described as causative genes for early-onset Alzheimer dementia (EOAD): *APP*, *PSEN1* and *PSEN2*. We describe a woman with EOAD had a novel *PSEN1* mutation.

**Case report:**

A 54-year-old right-handed woman presented 12-year history of progressive memory decline. She was clinically diagnosed as familial Alzheimer's disease due to a *PSEN1* mutation.

One of two daughters also has the same mutation, G209A in the TM-IV of PS1 protein. Her mother had unspecified dementia that began at the age of 40s. PolyPhen2 and SIFT prediction suggested that G209A might be a damaging variant with high scores. 3D modeling revealed that G209A exchange could result significant changes in the PS1 protein.

**Conclusion:**

We report a case of EOAD having probable novel *PSEN1* (G209A) mutation verified with structural prediction.

## Background

Alzheimer’s disease (AD) is the most common form of senile dementia, which could usually occur in patients over 60 years old. AD occurring at younger ages (early onset AD, EOAD) was quite rarely reported (5-10 % of all AD cases). Genetic background of EOAD was well-defined with three main genes: *amyloid precursor protein (APP), presenilin1 (PSEN1) and presenilin2 (PSEN2). PSEN1*, located on chromosome 14, was described as the most common causative gene for EOAD, due to the current reports more than 200 pathogenic mutations (AlzGene, www.alzgene.org). Majority of these mutations were associated with positive family history with autosomal dominant inheritance pattern. Several *de novo* EOAD cases were also reported, where no additional affected family members was found. The age of disease onset, associated with *PSEN1* mutations could be variable, and the most of the cases occurred at age of onset at 40–50 years old [1].

In this manuscript, we report a novel *PSEN1* G209A mutation in an EOAD patient. This mutation did not exist in any of the AD mutation databases, and it could be a novel causative mutation for EOAD.

## Case presentation

**Fig. 1:**
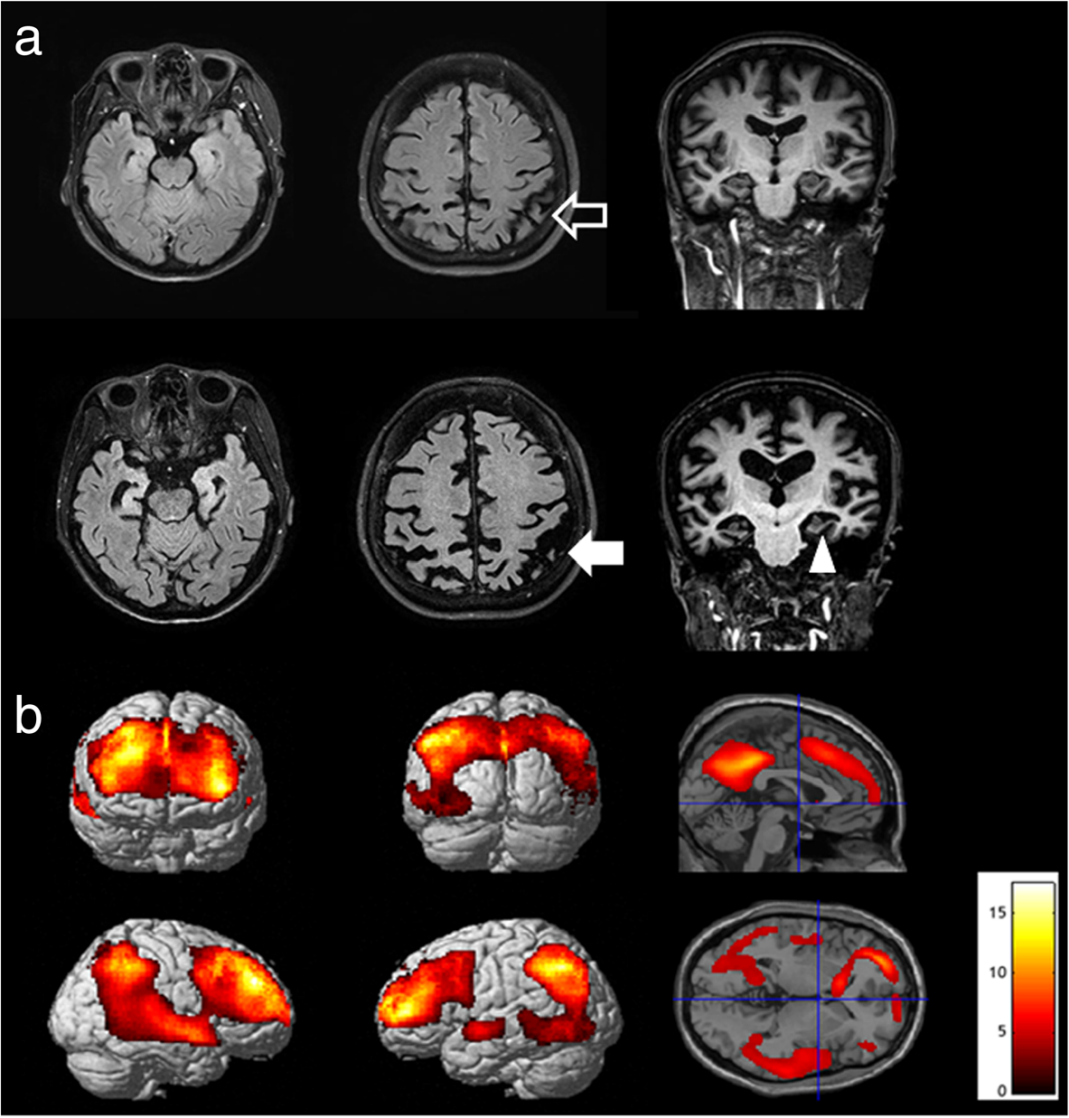
**a** Axial and coronal FLAIR images of brain MRI. The upper images at the age of 48 show that bilateral parietal atrophy (empty arrow). The lowers at the age of 54 show that diffuse cortical atrophy and prominent atrophic change in medial temporal (arrow head) and bilateral parietal lobes (filled arrow). **b** Statistical parametric maps (11 age matched control subjects; *p* < 0.001, uncorrected; extent threshold = 50 voxels) of FDG-PET of the patient, which reveal the bilateral temporal, parietal, precuneal and frontal hypometabolism. The color scale of statistical parametric maps indicates the Z value magnitude, with the lowest value appearing in dark red and the highest value in bright yellow/white

A 54-year-old right-handed woman presented 12-year history of progressive memory decline. She was a university educated woman. At the age of 42 years, her main symptoms were that she forgot names and needed to make notes. She had poorer recall of information than the others at her school of hotel management. In contrast, she was independent in all activities of daily living but her mood was depressed. At initial consultation, when she was 45 years old, general neurological exam was unremarkable. Attention, language and visuospatial function and frontal executive function were normal. Apraxia, dyscalculia and Gerstmann syndrome were not shown. However, Hopkin’s verbal learning test showed that free-recall score was 21 (5-8-8) and 20 min delayed-recall was 2. Its recognition score presented that true positive was 8 and false positive was 3. Delayed recall of Rey figure score was 5.5. Mini-Mental State Examination (MMSE) was 27/30, time orientation 4/5 and three word recall 2/3. Magnetic Resonance Imaging (MRI) scan are shown in Fig. 1 a. The upper axial and coronal FLAIR images showed that medial temporal lobe atrophy was not prominent but bilateral parietal atrophy was on. Her *APOE* genotype was homozygous for ε3. Her mother had dementia at the age of 40s and died before 60s. No specific diagnosis was made in her mother. Her father and only brother did not show dementia. She has two daughters, 29 and 26 years old. The initial clinical impression was mild cognitive impairment with depression. However, AD could not be ruled out. She showed progressive deterioration in verbal and visual memory. At the age of 53, her MMSE was 2/30 and clinical dementia rate (CDR) was 3. She was able to respond only to her name and distinguish her daughter from unfamiliar people. She needed personal care. Her brain MRI (Fig. 1 a, lower) demonstrated that global cortical atrophy, more prominent in medial temporal and parietal lobes. FDG-PET (fludeoxyglucose- positron emission tomography) of the patient reveals the bilateral temporal, parietal, precuneal and frontal hypometabolism (Fig. 1 b). They consent to perform genetic sequencing on her and her 2 daughters. We screened the *APP, PSEN1, PSEN2* and *PRNP (prion protein)* gene.

### Genetic analysis of *PSEN1* and structural prediction of mutant PSEN 1 protein

#### Methods

White blood cells (or buffy coat) were separated by centrifugation at 800 g (30 minutes). Genomic DNA was extracted by GeneAll blood kit (Seoul Korea) as described in the protocol. We performed a polymerase chain reaction (PCR)-based genetic analysis. We used PCR primers for *APP* exon 16 [2] and 17 [3], *PSEN1* exon 4, 5, 6, 7, 8 and 11 [4, 5], *PSEN2* exon 4, 5, 6, 7 and 12 [4] and *PRNP* genes [6]. Single-strand conformation polymorphism (SSCP, Fig. 2 a) analysis was performed with the PCR products. Denaturation buffer was added to the amplicons (50:50), which was composed of 95 % formamide, 18mMol EDTA and 0.025 % xylene cyanol, and bromophenol blue. These mixes were incubated on 98 °C for 10 minutes, resulting in the denaturation of double stranded DNA bands [7]. The single stranded DNA bands were separated in non-denaturing polyacrylamide gel electrophoresis (PAGE) (10-12 %) for 18–21 hours (BioRAD, Seoul, Korea). Depending on the mutations, single stranded DNAs could have different mobility in the gel. SYBR Gold staining (Invitrogen, USA) was used for staining DNA bands, which visualized under UV light. To confirm and identify the mutation, sequencing was performed for all PCR products at both directions by the BioNeer Inc. (Dajeon, Korea). Prior to sequencing, we purified the PCR products by GeneAll PCR protocol kit (Seoul, Korea), as described in the protocol. For the sequencing reactions, Big Dye Terminator Cyclic sequencing was performed on ABI 3730XL DNA Analyzer (http://eng.bioneer.com/home.aspxURL: Please check that the following URLs are working. If not, please provide alternatives: http://eng.bioneer.com/home.aspxYes, we have checked and it was working well., Bioneer Inc., Dajeon, Korea) was used (Fig. 2 b and c). We aligned the sequenceing results by NCBI Blast (http://blast.ncbi.nlm.nih.gov/Blast.cgiURL: Please check that the following URLs are working. If not, please provide alternatives: http://blast.ncbi.nlm.nih.gov/Blast.cgiYes, we have checked and it was working well.), and the chromatograms were analyzed by DNA BASER (http://www.dnabaser.comURL: Please check that the following URLs are working. If not, please provide alternatives: http://www.dnabaser.comYes, we have checked and it was working well.) software. Mutations and sequence variants were identified by using the NCBI Gene (http://www.ncbi.nlm.nih.gov/geneURL: Please check that the following URLs are working. If not, please provide alternatives: http://www.ncbi.nlm.nih.gov/geneYes, we have checked and it was working well.) and UniProt (http://www.uniprot.orgURL: Please check that the following URLs are working. If not, please provide alternatives: http://www.uniprot.orgYes, we have checked and it was working well.) databases.

**Fig. 2Figure: Upon checking, it was noticed that there are panels in the caption of Figure 2; however, panel "c" was not found in the corresponding image. Please provide us with an updated figure with corresponding panels matching their description in the figure caption.Thank you for your suggestion. 'c' was changed to 'Sample'. We also change the 'a' to 'A' and 'b' to 'B'.**
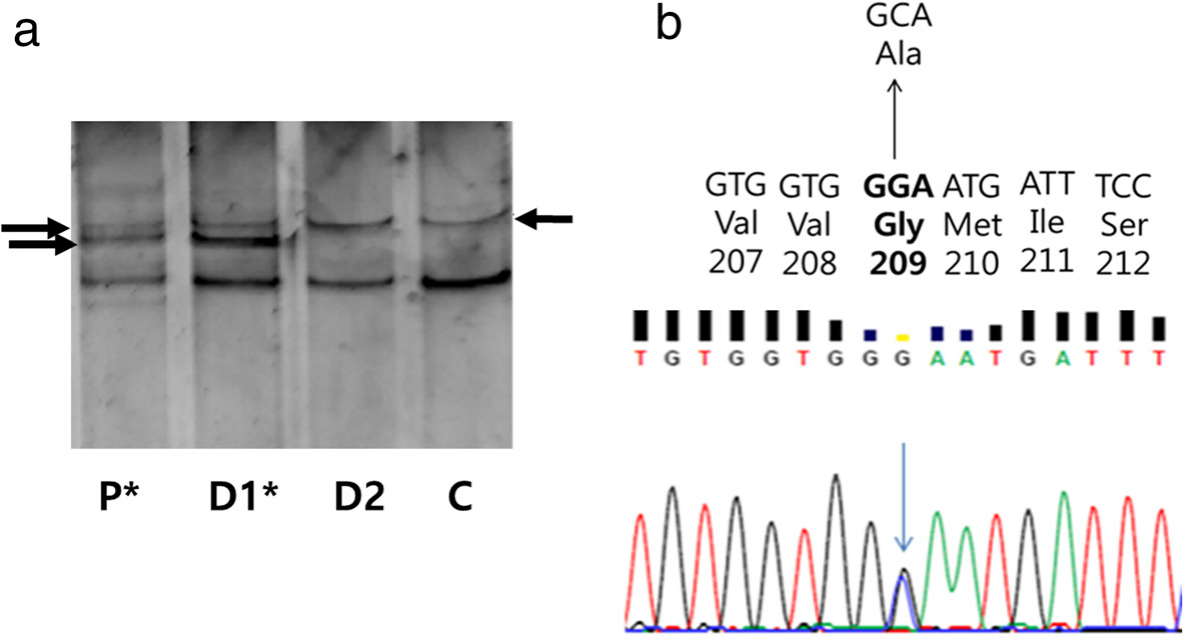
**A** SSCP data for *PSEN1* G209A. **Sample** was verified as negative for any mutation in *PSEN1* exon7. P*, proband; D1*, affected daughter; D2, unaffected daughter; C, control sample. **B** Sequencing data of EOAD patient with *PSEN1* G209A and location of G209A in *PSEN1*

Mutations were analyzed by an online software, the Polymorphism Phenotyping v2 (PolyPhen 2, http://genetics.bwh.harvard.edu/pph2/URL: Please check that the following URLs are working. If not, please provide alternatives: http://genetics.bwh.harvard.edu/pph2/Yes, we have checked and it was working well.), which could make *in silico* predictions on the putative damaging properties of mutations. This software made a comparison on the wild-type and mutant alleles of proteins by homology searching and multiple sequence alignment. Identity- and structure-based searches, such as accessible surface area and hydrophobicity features, were also performed. These predictions might define the potential role of amino acid exchanges in the proteins. Three categorized of mutations could be available: probably, possibly damaging or benign variants. PolyPhen2 could also provide a multiple sequence alignment, by comparing the homologous sequences from different vertebrate and non-vertebrate species. Two types of datasets could be available: the HumDiv and HumVar scores. HumDiv should be used for the prediction of putative pathogenic nature of rare alleles. In HumDiv scores, the alleles with mildly damaging properties should be categorized as possibly damaging. HumVar should be used in case of diagnosis of Mendelian disorders. Here, the highly damaging phenotypes should be distinguished from the alleles with less damaging phenotypes [8].

Sorting Intolerant from Tolerant or SIFT algorithm (http://sift.jcvi.org/) is an online software, which estimates the potential effects of missense mutations substitution on the protein function. SIFT uses several protein databases, including SWISS-PROT, SWISS-PROT/TrEMBL, or protein databases of NCBI, and calculates the possibility on the damaging nature of mutations by the comparison of the mutant and normal proteins. It scores the amino acid substitutions, and under and over the score of 0.05, mutations could be defined as deleterious or tolerated, respectively diction [9].

**Fig. 3:**
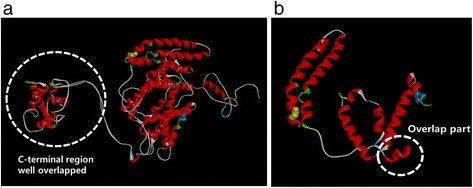
3D model modeling for PS1 G209A. Differences of normal and mutant PSEN1 have been highlighted in the black circle, and glycine was labeled with yellow, while alanine was labeled with yellow. The C-terminal pert of normal and abnormal proteins were overlapped, while significant differences could be seen in the N-terminal part, suggesting that the mutation could result significant conformational changes in the TM structure of PSEN1. **a** Differences between normal PSEN1 and PSEN1 G209A, focused on all over the protein. **b** Differences between normal and mutant PSEN1, focused on the TM-IV region.

3D structures of PS1 with mutation(s) were analyzed by an online software, the Raptor X (http://raptorx.uchicago.edu/URL: Please check that the following URLs are working. If not, please provide alternatives: http://raptorx.uchicago.edu/Yes, we have checked and it was working well., Fig. 3). Server of protein structure prediction used the full amino acid sequence of PS1 (1–467 amino acids). Discovery Studio 3.5 Visualizer from Accelrys was used to display the images after superimposition [10].

### Results

Alternative mobility was found in the SSCP (Fig. 2 a) for exon 7 of *PSEN1* gene. Sequencing revealed a G → C exchange at codon 209, resulting in a glycine (GGA) → Alanine (GCA) exchange (Fig. 2 b and c). This mutation was not found in neither in the AD and FTD mutation database nor in the Alzgene database, which suggested that it might be a novel mutation in *PSEN1*. Mutation was checked in the Exome Aggregation Consortium (ExAC) database, which screened 60,706 unrelated individuals for the different disease causing genes (http://exac.broadinstitute.org/URL: Please check that the following URLs are working. If not, please provide alternatives: http://exac.broadinstitute.org/Yes, we have checked and it was working well.). *PSEN1* G209A was not found in this database.

PolyPhen-2 scores were high, both HumDiv and HumVar was 1.000 (sensitivity: 0.00; specificity: 1.00 at both scores) suggesting G209A as a probably damaging variant. Multiple sequence alignment suggested that glycine 209 might be a conservative residue among vertebrates. Several mammalian (such as dogs, pigs) and non-mammalian (such as chicken, frog, zebrafish) showed glycine at the same position in their presenilin or presenilin-like proteins. SIFT prediction also confirmed the probably damaging nature of mutation, with the score of 0.

3D prediction (Fig. 3) revealed significant differences in the N-terminal region of normal PS1 protein. The c-terminal region of normal PS1 and PS1 with G209A were overlapped. These findings suggest that even glycine and alanine are both small sized, hydrophobic amino acids, but one extra –CH3 group on alanine could result significant differences in the protein structure. Since alanine is more hydrophobic than glycine, it could result extra stress inside the helix.

### Discussion

We reported a novel mutation in *PSEN1*, the G209A in a female patient in her early 50s. Even though we could not find any affected family members, one of her daughters is a mutation carrier and deceased mother had an undiagnosed case of dementia. Our finding suggested that this mutation could be a novel probably pathogenic variant, involved in EOAD.

Glycine 209 is located in the transmembrane –IV region of PS1 protein, and it was suggested as a conservative residue. For codon 209, three additional mutations were found, G209R, G209E and G209V (Table 1), which also suggested that Glycine 209 might be an important residue in PS1. G209R was found in a Japanese family. The proband was a male patient, the disease progression started at the age of 46. Additional affected family members were found, since his sister and mother also showed clinical symptoms. All of these patients showed rapid progression of dementia, with memory impairment, amnesia, aphasia, disorientation and personality changes. Parietal focal symptoms such as apraxia or agnosia were not apparent in the patients [11]. G209V was found in a German family, where the age of onset ranged between 30–48 years. Clinical symptoms included myoclonus, seizures, language loss and apraxia and agnosia [12, 13]. G209E was reported in one AD patient by Rogaeva. No detailed information was found on the disease age of onset or clinical phenotype of disease, associated with G209E [14].

**Table 1:** Comparison of G209R, G209E, G209V and G209A

Mutation	Gly209Arg	Gly209Glu	Gly209Val	Gly209Ala
Age of onset	46 – 53 years	unknown	30 - 48 years	54 years
Age of death	unknown	unknown	unknown	Surviving
Family history	positive	Unknown (detected in 1 patient)	positive	Unknown (since her mother had undiagnosed early-onset dementia)
Clinical phenotype	Rapid progressive dementia characterized by memory impairment, amnestic aphasia, disorientation and personality change, but lacking parietal focal symptoms such as apraxia or agnosia	unknown	AD with myoclonus, seizures, language loss, aphasia	Mild cognitive impairment with depression, followed by progressive deterioration in verbal and visual memory
Reference	Sugiyama et al. 1999 [11]	Rogaeva et al. 2001 [14]	Poorkaj et al. 1998 [12] Larner et al. 2006 [13]	

In silico modeling and the previously described mutations for G209 could support that *PSEN1* G209A could be a novel causative mutation for AD. Glycine and alanine are similar, both of them are small sized hydrophobic amino acids. 3D prediction suggested that in this mutation, a single extra –CH3 group could result disturbances inside the PS1 protein. Since alanine is more hydrophobic than glycine, it could enhance the stress inside the PS1 TM domain or between PS1 and its binding partners.

As conclusion, we suggest that G209A can be a novel, probably pathogenic variant in *PSEN1*. Guerrio et al. published an algorithm for the novel mutations in *PSEN1* and *PSEN2*. Several criteria were analyzed: such as whether the mutation is segregates with the disorder segregation; whether there are additional affected family members; and whether additional mutations were described for the screened residue [15]. *PSEN1* G209A was not found in the ExAC database, which could be a strong evidence on its pathogenic nature. The additional three, probably pathogenic mutations for the glycine at position 209 (G209R, G209V and G209E) can also enhance the importance of residue 209 in *PSEN1* and the probably damaging nature of G209A. However, the family history of patient could not be well-defined. Her mother was a dementia patient, but it remained undiagnosed, so it is unclear whether she is the carrier of mutation. One of her daughter carried the mutation, who might be in the pre-symptomatic stage of disorder. Online predictions were suggested that *PSEN1* G209A mutation could be a probably deleterious variant. Additional studies are needed on for this variant since its pathogenic nature can’t rely on the in silico predictions only.

## Conclusions

We suggest that G209A can be a novel, probably pathogenic variant in *PSEN1*. Combination of clinical-, association studies, prediction- and 3D modeling software can enhance the estimation on the pathogenic nature of novel variants.

### Consent

Written informed consent was obtained from the legal guardian of patient (her daughter) for publication of this Case report and any accompanying images. They also consent to perform genetic sequencing on their mother and themselves. A copy of the written consent is available for review by the Editor of this journal.Additional file: Checklist was received as additional file. Please confirm if indeed the file is appropriate to be captured as additional file. If yes, please provide its corresponding citation in the main text. Please note that additional files should be cited in ascending numerical order in the main body of the text.Otherwise, please advise if we will delete the said file.Dr. Youn will answer this query.

## Abbreviations

AD: Alzheimer’s disease
EOAD: Early-onset Alzheimer’s disease
MMSE: Mini-mental state examination
MRI: Magnetic resonance imaging
FDG-PET: Fludeoxyglucose- positron emission tomography
TM-IV: Transmembrane segment IV
PS1: Presenilin-1
APP: Amyloid precursor protein
PSEN1: Presenilin 1
PSEN2: Presenilin 2
PRNP: Prion protein
PCR: Polymerase chain reaction
PAGE: Polyacrylamide gel electrophoresis
PolyPhen2: Polymorphism phenotyping v2
SIFT: Sorting intolerant from tolerant
